# Polymer-Rich Dense Phase Can Concentrate Metastable Silica Precursors and Regulate Their Mineralization

**DOI:** 10.1021/acsbiomaterials.2c01249

**Published:** 2023-02-01

**Authors:** Hang Zhai, Yuke Fan, Wenjun Zhang, Neta Varsano, Assaf Gal

**Affiliations:** † Department of Plant and Environmental Sciences, Weizmann Institute of Science, Rehovot 7610001, Israel; ‡ College of Resources and Environment, Huazhong Agricultural University, Wuhan 430070, China; § Department of Chemical Research Support, Weizmann Institute of Science, Rehovot 7610001, Israel

**Keywords:** coacervation, bioinspired materials, silicification, mineralization pathways

## Abstract

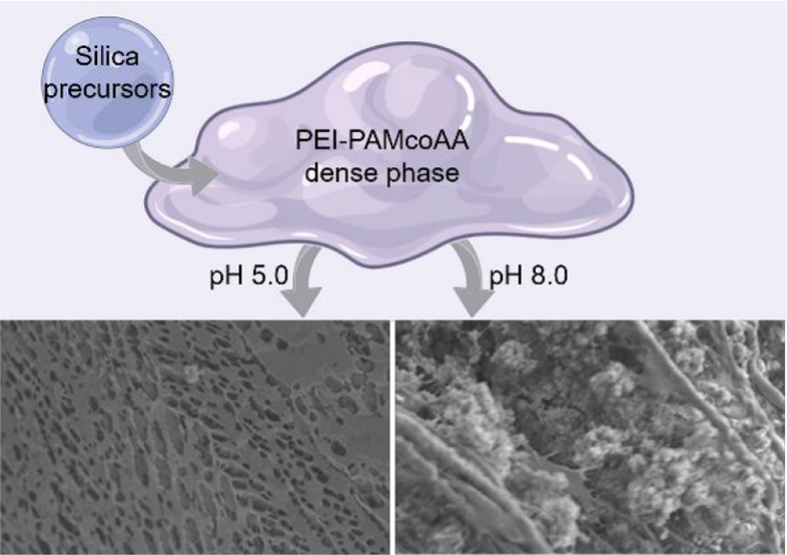

Multistep mineralization processes are pivotal in the fabrication of functional materials and are often characterized by far from equilibrium conditions and high supersaturation. Interestingly, such ‘nonclassical’ mineralization pathways are widespread in biological systems, even though concentrating molecules well beyond their saturation level is incompatible with cellular homeostasis. Here, we show how polymer phase separation can facilitate bioinspired silica formation by passively concentrating the inorganic building blocks within the polymer dense phase. The high affinity of the dense phase to mobile silica precursors generates a diffusive flux against the concentration gradient, similar to dynamic equilibrium, and the resulting high supersaturation leads to precipitation of insoluble silica. Manipulating the chemistry of the dense phase allows to control the delicate interplay between polymer chemistry and silica precipitation. These results connect two phase transition phenomena, mineralization and coacervation, and offer a framework to achieve better control of mineral formation.

## Introduction

Formation of inorganic materials from soluble building blocks is fundamental to biological systems and technological applications alike.^1,2^ It is established that in addition to the classical monomer-by-monomer growth process, a myriad of multistep alternatives exist.^3^ A common feature of these ‘nonclassical’ mineralization processes is that they occur at conditions that are far from equilibrium and often involve additives that serve as process-directing agents.^4^ In synthetic systems, highly supersaturated solutions are attainable by mixing concentrated solutions or using rapid chemical reactions that accumulate products. These conditions are usually incompatible with biological processes that need to occur within the general homeostasis of the cell.^1^ Therefore, when considering the two hallmarks of multistep mineralization, a crowded environment and high supersaturation, they match very differently common cellular settings. On the one hand, the cellular environment is inherently crowded with functional macromolecules, but on the other hand, it is difficult to envision how cells concentrate the mineral building blocks to the needed supersaturation values allowing the formation of metastable phases.

On a wider perspective, controlling chemical reactions is a fundamental trait of biological systems, and organisms use various strategies to regulate where and when to activate a desired chemical process. One such strategy is the use of intracellular condensates, dense biopolymer phases that form through the physical process of liquid–liquid phase separation.^5,6^ These condensates create distinct chemical conditions, which localize chemical reactions to specific environments within the different phases.^7^ One outcome of the different chemical and physical properties within the dense condensate and in the surrounding dilute phase is that concentration gradients appear for ‘client’ molecules that diffuse freely between the dense and dilute phases to reach equilibrium.^8−10^ The magnitude of such a gradient and its direction is highly dependent on the specific chemistry of the system.

It is attractive to study the involvement of liquid–liquid phase separation in mineralization processes, as both phenomena fundamentally involve phase transitions.^11−17^ One of the most intriguing examples is the formation of silica at physiological conditions within cells,^18^ a process that is extremely different from the harsh chemical conditions that are used in industrial silica applications.^19^ A hallmark of biogenic silicification processes is the presence of oppositely charged polymers, cationic long-chain polyamines, and negatively charged proteins, that can phase separate, forming a dense polymer-rich phase, or a coacervate, within a dilute matrix.^20−22^ Several bioinspired silicification experiments suggested that liquid–liquid phase separation is involved in various stages of the process,^23−25^ albeit not as a mandatory feature.^26^ But even though it was recently demonstrated that the polymer dense phase creates a distinct chemical environment that facilitates the formation of dense silica particles,^27^ the microscopic size of the dense phase droplets precluded the ability to elucidate the chemistry that leads to the regulated silicification process.

Here, we use a synthetic system of macroscopic phase separation to investigate the mechanism of silica formation within dense polymer phases. By following the kinetics of silica diffusion between the dense and dilute phases, we give quantitative description of the condensate-mediated silicification. We show that the dense phase can concentrate mobile silica species, which then polymerize at appropriate conditions. This opens the option to replace the current harsh chemical conditions for producing silica-based materials with bioinspired routes.

## Results

**Figure 1:**
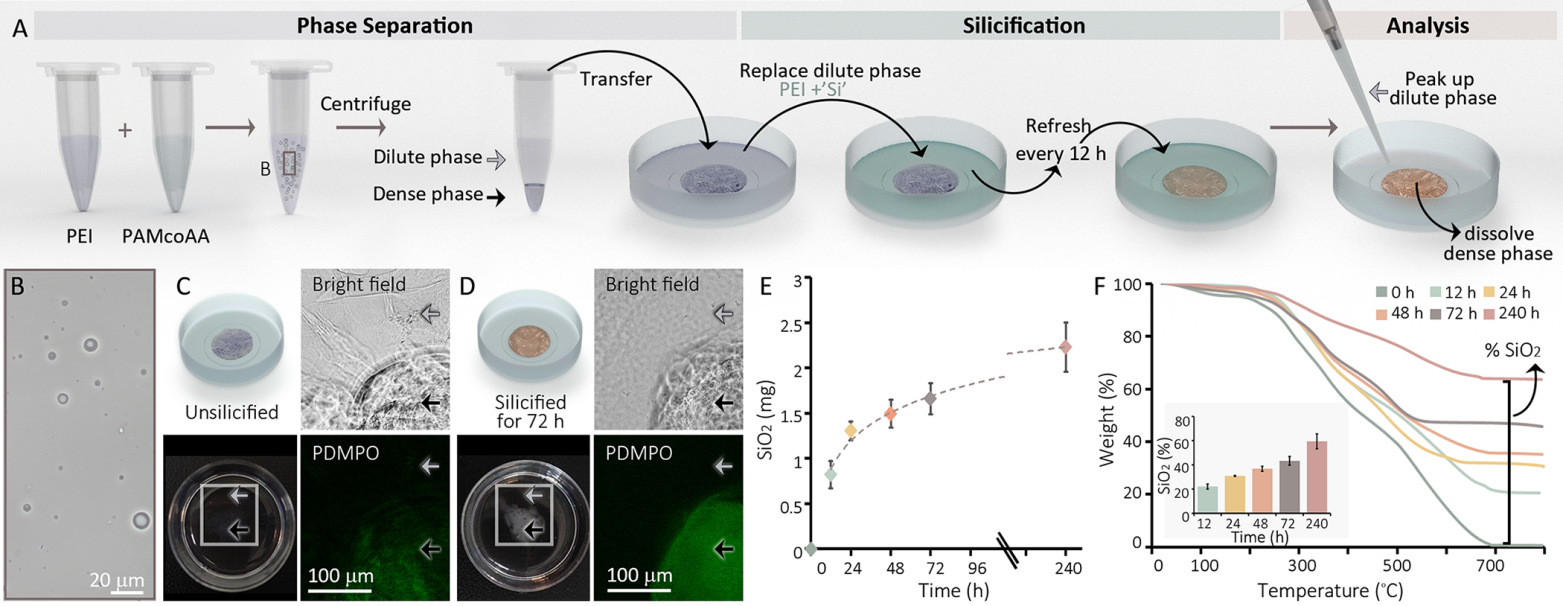
A polymer phase separation system that concentrates silica in the dense phase. (A) The experimental pipeline consisting of three stages. (B–D) Light microscopy images of the various stages of the process. Note that the macroscopic dense phase in C and D concentrates the silica tracker dye PDMPO (green) when silica precursors are introduced to the dilute phase. (E) The amount of silica inside the polymer dense phase after incubation with a dilute phase containing 100 mM dissolved Na_2_SiO_3_ in PEI. (F) Analyses of the amount of incombustible inorganic content (mainly silica) present in lyophilized dense phases.

A major limitation for silicification studies of many established liquid–liquid phase separation systems is that the micrometer-scale droplets of the dense phase are dispersed in the surrounding dilute phase.^22,23,27^ This precludes the use of bulk analyses for the study of dynamic interactions between the dense and dilute phases. We explored various combinations of positively charged, amine-containing, and negatively charged polymers that will yield macroscopic phase separation. Such system was achieved by mixing 50 mM of polyethylenimine (PEI) and poly(acrylamide-*co*-acrylic acid) (PAMcoAA) (Figures S1 and S2). Immediately after mixing the positively charged PEI and the negatively charged PAMcoAA, the solution became turbid due to the formation of dense coacervate droplets. Letting the droplets settle or using mild centrifugation led to the coalescence and fusion of the droplets into a single dense phase (Figure 1A, B).

In order to study silicification in this system, the two phases were transferred to a Petri dish where the dense phase could spread on the hydrophilic surface, thus maximizing its surface area and reducing the time needed for diffusion between the phases. Once in the Petri dish, the original, thermodynamically equilibrated, dilute phase was replaced with a Si-containing dilute solution (Figure 1A). The new dilute phase was a sodium silicate solution of various concentrations stabilized by 10 mM PEI. In these solutions, the PEI can stabilize most of the supersaturated silicate species from gelation for a few days by the formation of various oligomers,^28^ while the concentration of monomeric silicic acid is very close to the saturation value (Figures S3 and S4). Because the dilute phase in many of our experiments contains most of its silica content as various oligomeric structures, we will refer to them collectively as ‘Si’. In our experimental setup, the new dilute phase was refreshed every 12 h to avoid macroscopic gelation and facilitate experiments spanning several days. A qualitative examination of this system shows that in the absence of Si both dilute and dense phases are transparent, but after introducing the Si-containing dilute phase, the dense phase changes its appearance to opaque and accumulates the fluorescent dye PDMPO that has high affinity to forming silica (Figure 1C, D).^29^ Therefore, our system enables to follow a coacervate induced silicification process with the ability to differentiate between the dense and dilute phases.

We monitored the kinetics of coacervate silicification by conducting such experiments for time periods ranging from 12 h to 10 days. At the end of the experiment, the dilute phase was removed and the dense phase was fully dissolved in a strong base (Figure 1A). The amount of ‘Si’ extracted from the dense phase was quantified with a colorimetric method.^30^ These experiments show a logarithmic increase in the ‘Si’ content of the dense phase, resembling a dynamic equilibrium (Figure 1E). The silicification of the dense phase was quantified also by a thermogravimetric analysis (TGA) of lyophilized ‘Si’-containing dense phases, demonstrating silica content that rises from 0% to ∼60% dry weight after 10 days (Figure 1F). These results demonstrate that mobile ‘Si’ species diffuse from the dilute phase and accumulate with time in the dense phase.

**Figure 2:**
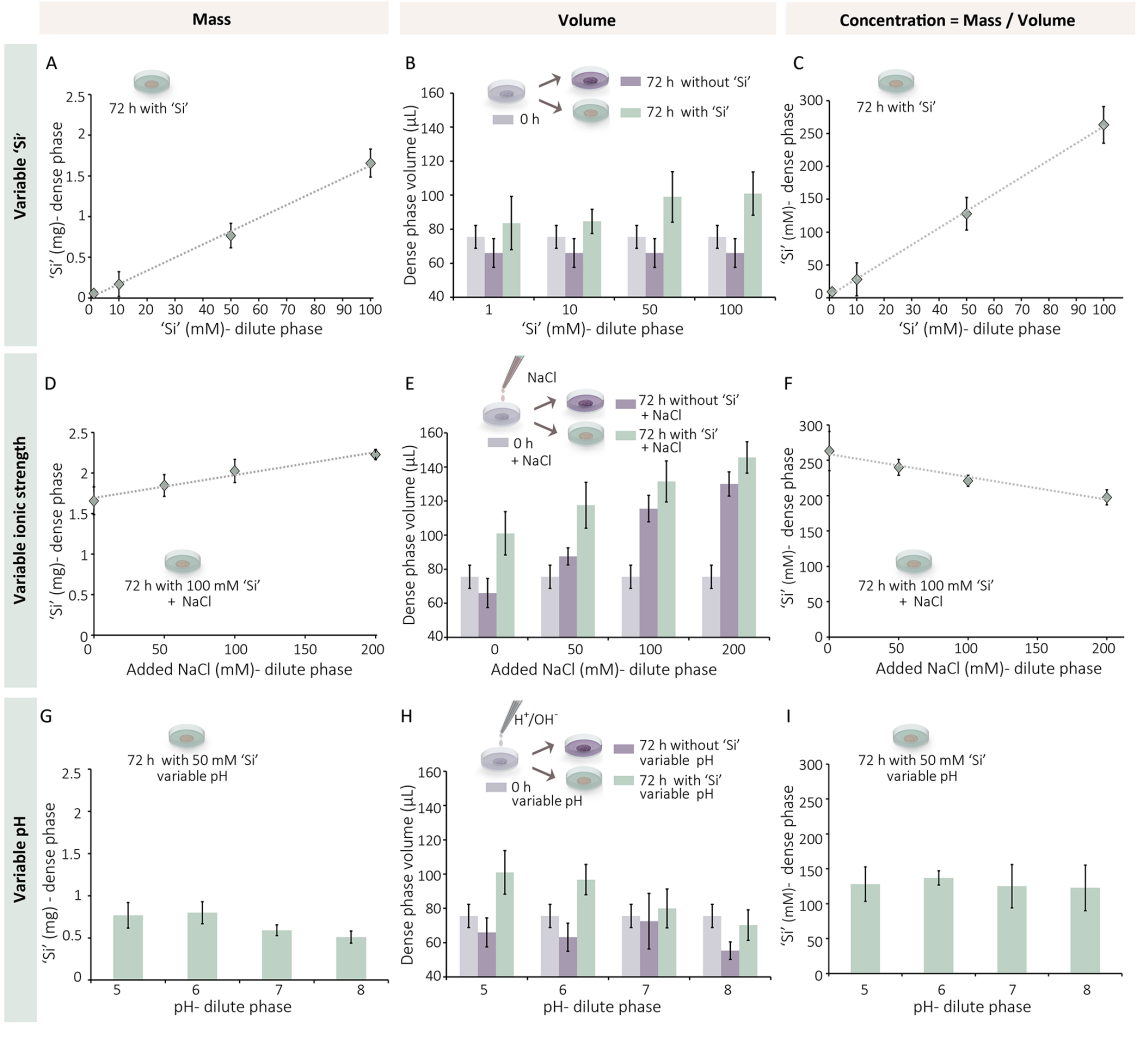
Si concentrations within the polymer dense phase. (A–C) Experiments of dense phase silicification with variable ‘Si’ concentrations in the dilute phase. (A) The measured amount of silica extracted from the dense phase after 72 h and (B) its volume are used to calculate (C) the concentration of silica in the dense phase. (D–I) Similar experiments with a constant Si concentration and (D–F) variable ionic strength or (G–I) pH of the dilute phase. As in all cases, we do not know the exact chemical state of the silica species we refer to them collectively as ‘Si’.

We used our experimental system to explore how the concentration of dissolved Na_2_SiO_3_ in the dilute phase affects the amount of silica accumulated in the dense phase. The silica quantity in the dense phase was measured after 72 h incubations with the ‘Si’-containing dilute phase. The results showed a linear relation between ‘Si’ concentration in the dilute phase and silica quantity in the dense phase (Figure 2A). In order to calculate the concentration of Si in the dense phase we measured its volume at the various conditions. These measurements show a mild volume reduction of the dense phase in the absence of ‘Si’, and an increase of the volume in the presence of ‘Si’ (Figure 2B, Figure S5). These volume changes are due to shifts toward new equilibrium of the polymer phase separation system, induced by changing the original composition of the dilute phase with the new Si-containing solution. We used the quantity of ‘Si’ in the dense phase and its volume to calculate the ‘Si’ concentration in the dense phase after 72 h. This shows that ‘Si’ concentrations follow a similar trend to that of ‘Si’ quantities, namely, an increase that is linearly correlated to ‘Si’ concentration in the dilute phase (Figure 2C). Remarkably, even though the trends are similar, the nominal concentrations in the dilute and dense phases are very different, with concentrations of ‘Si’ in the dense phase higher by a factor of ∼2.5. This demonstrates an uptake process that seems to proceed against a concentration gradient without any active energy-driven process.

We evaluated the influence of other factors that are known to influence silica formation,^27,31^ on the concentrating ability of the dense phase. Elevating the ionic strength of the dilute phase by adding NaCl had a clear effect of expanding the volume of the dense phase (Figure 2E). This is expected due to diffusion of ions into the dense phase, contributing to charge screening that will loosen the intermolecular attraction and lead to a more hydrated dense phase.^32,33^ In addition, the quantity of silica rose with added salt, although when calculating ‘Si’ concentration a decrease was observed (Figure 2D, F). This suggests that the expansion effect is dominant, allowing some additional ‘Si’ uptake as a byproduct, but the efficiency of ‘Si’ uptake is best without added salt. Changing the pH value of the ‘Si’-containing dilute phase also affected the system. Elevating the pH countered the expansion of the dense phase in the presence of ‘Si’, which together with a reduction in the amount of ‘Si’ leads to a constant ‘Si’ concentration in the dense phase (Figure 2G–I). Altogether, these observations point to a complex interaction landscape between the phase separating polymers and the silica species introduced in the dilute phase. The dense phase clearly serves as a sink for high concentrations of silica but the balance between the two phases can be manipulated by other factors such as ionic strength and pH.

**Figure 3:**
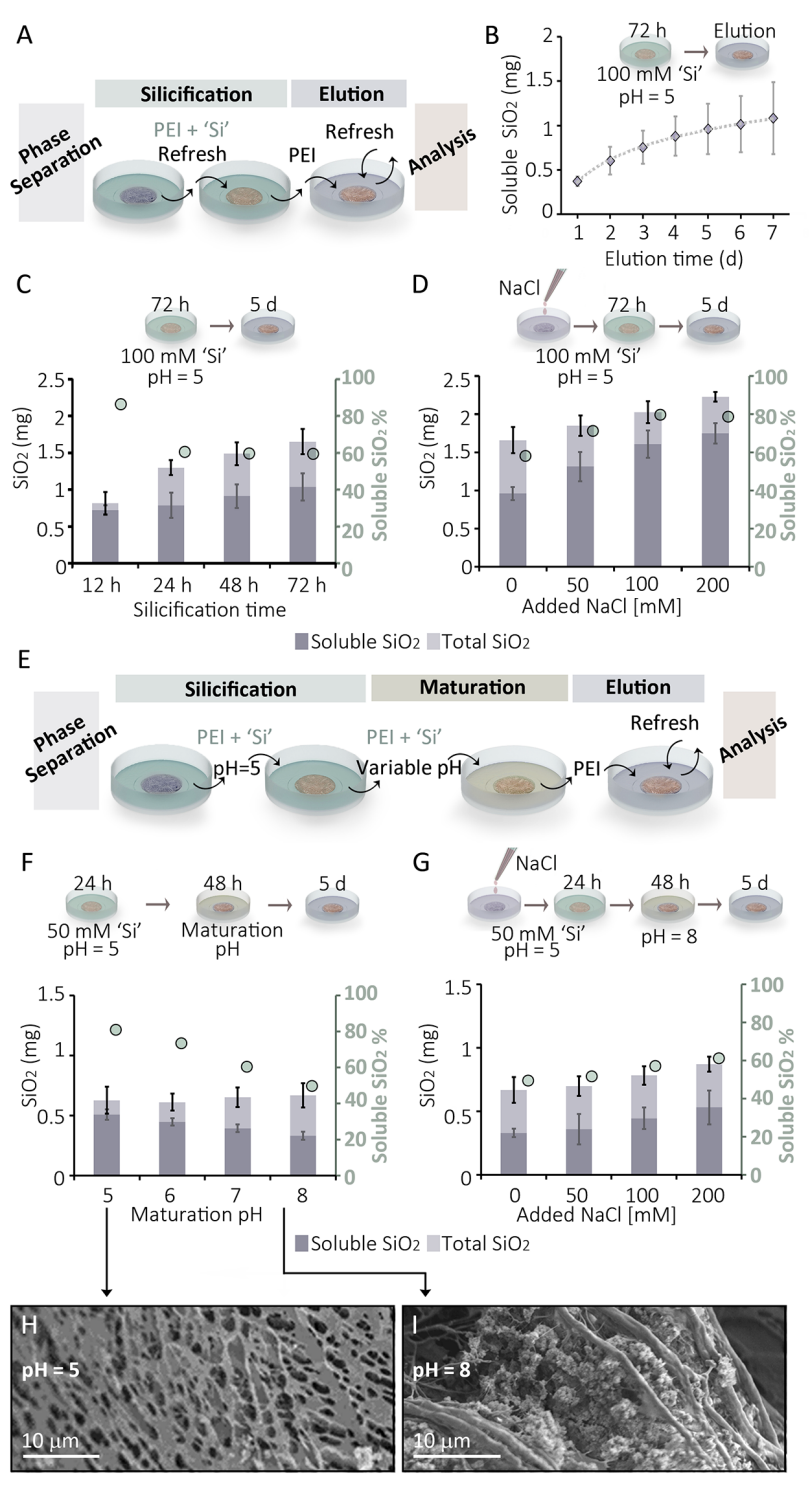
Precipitation of insoluble silica inside the dense phase. (A) The modified experimental setup where an elution step is added to measure the amount of soluble silica that can diffuse out of the dense phase. (B) The cumulative amount of eluted silica, measured every 24 h when refreshing the Si-free dilute phase. (C) The amount of soluble silica (eluted during 5 days), and total silica after various silicification times. The %soluble is plotted by the floating markers. (D) The amount of soluble silica as a function of ionic strength in the dilute phase. (E) A further modification to the experimental setup where the silicification step is divided into two steps with different pH. (F) The amount of soluble silica (eluted during 5 days) and total silica after silicification with various pH changes. (G) The amount of soluble silica at pH 8 as a function of ionic strength in the dilute phase. (H, I) SEM images of freeze-dried silicified samples following maturation under different pH values.

The ability of the dense phase to concentrate such high amount of ‘Si’ from the dilute phase brings the question of the chemical driving force. It is possible that inside the dense phase the silica matures into an insoluble and immobile phase that is inert to diffusion, thus allowing continuous inward diffusion of fresh mobile silica. However, the regular ratio between ‘Si'’ concentrations in both phases (Figure 2A) is reminiscent of a system with a fractionation coefficient that reaches dynamic equilibrium, where both silica pools are mobile and take part in the chemical equilibrium. We investigated this scenario by adding an elution step after the silicification step (Figure 3A). For elution, after the designated silicification period, the ‘Si’-containing dilute phase was replaced with an identical, but ‘Si’-devoid, dilute phase. The amount of soluble silicic acid released into the dilute phase was measured each time it was refreshed. The data show a time dependent increase in the cumulative amount of eluted soluble silica from the dense phase, as expected in a ‘salting-out’ system (Figure 3B). We term the sum of all soluble silica eluted within 5 days as the amount of mobile silica in the dilute phase (Figure S6). By dissolving the dense phase in parallel experiments directly after the silicification step, we quantified the total amount of silica within it and could calculate the amount of insoluble silica.

The results show a gradual decrease in the fraction of soluble silica with time (Figure 3C). Initially, after only 12 h of silicification, almost all the silica in the dense phase is in a diffusible state. As the silicification period is extended, more silica is found within the dense phase and its insoluble fraction is also growing. Additional experiments using *in situ* Raman spectroscopy to characterize the silica phases inside the dense phase confirmed a gradual polymerization with time (Figure S7). This suggests that alongside the diffusion of silica into the dense phase, polymerization is occurring. However, polymerization cannot be the driving force for silica diffusion into the dense phase as the amount of mobile silica by itself increases with time, and we therefore conclude that uptake is controlled by diffusion, while time-dependent polymerization is superimposed on this process. Another evidence supporting this conclusion is that direct measurements of insoluble silica by dissolving the dense phase after elution, constantly gave values that are smaller than the difference between total and mobile silica (Figure S8), making the calculated mobile fraction an underestimate. This is probably because of oligomeric species that diffuse into the dilute phase but are undetectable by the colorimetric method (Figure S6).^30^

Varying chemical conditions of the phase separation system can affect the fractionation between the two phases (Figure 2). We further investigated if there is also an effect on the extent of polymerization. Indeed, the ratio between mobile and insoluble silica can be controlled by ionic strength, where more added salt leads to a higher mobile silica fraction (Figure 3D). We tried to deconvolve diffusional fractionation between the two phases and the precipitation of silica within the dense phase. To this end, changing pH can be useful as it affects silica polymerization,^31^ but does not influence the concentration ratio between the phases (Figure 2I). For these experiments, the 72 h silicification period was divided in two. For 24 h, silica was introduced at pH 5, where it is most soluble, but for the following 48 h the pH of the dilute phase was changed to higher pH values, triggering a concomitant change in conditions inside the dense phase (Figure 3E). These experiments show that indeed the total amount of silica in the dense phase was constant, but the fraction of mobile silica decreased with higher pH values (Figure 3F). In addition, imaging freeze-dried samples of the silicified dense phase shows that high pH values result in granular silica deposits between the dried polymer networks (Figure 3H, I, Figure S9). The fine-tuned chemical interplay within the dense phase can be further demonstrated by contrasting the effect of high pH (the case of pH 8 in Figure 3F) with the opposite effect of added salt. Such experiments, where silica maturation was done at pH 8 with varying ionic strength, show that the added salt allows more silica in the dense phase and hinders its polymerization (Figure 3G). Altogether, silica polymerization occurs inside the dense phase to an extent and rate that is controlled by various chemical factors.

## Discussion

Previously, it was demonstrated that polymer phase separation has a regulatory role in silica formation as it creates a distinct environment within the dense phase.^27^ The present study shows that an inherent feature of the polymer dense phase is a higher affinity for soluble silica, which can facilitate the localized polymerization process in the dense phase. This lays a mechanistic framework where the dense phase passively concentrates mobile silica from the dilute phase due to diffusional dynamic equilibrium, and these metastable silica precursors preferentially precipitate inside the dense phase.

This framework explains some of the differences between silica polymerization within the dilute and dense phases. In the dilute phase, supersaturated silica precursors will undergo the known sol–gel process yielding a silica gel.^31,34^ On the other hand, the process of polymerization inside the dense phase occurs within a crowded environment in the presence of a higher silica concentration that is maintained via the dynamic equilibrium between the two phases. The outcome is the formation of silica granules within the organic matrix (Figure 3H, I, Figure S9).^27^

We circumvent the challenges of studying microscopic dense phase droplets within a dilute bulk phase by using a macroscopic phase separation system that allows to handle separately the dense and dilute phases. Nevertheless, this system suffers the limitation that diffusion gradients within the dense phase cannot be ignored. For example, when silicification was attempted inside a narrow Eppendorf tube, only the first few millimeters of the dilute phase closest to the interface showed visible mineralization. We addressed this issue partially by spreading the dense phase, thus enlarging its surface area, but its thickness was still large enough to expose morphological differences between the dense phase periphery and interior (Figure S10).

These inhomogeneities within the dense phase further complicate the interplay between the two important chemical processes, diffusion of mobile silica driven by dynamic equilibrium and the polymerization of insoluble silica species. Ideally, silica polymerization should consume all mobile silica in the dense phase and will support a continuous flux from the pool of mobile silica in the dilute phase until the entire dense phase will become silicified. However, the dense phase in our experiment only reaches ∼60% silicification. A plausible reason is that the gradients in the dense phase caused faster silicification of its periphery, disconnecting the interior from diffusional supply and creating an overall core–shell architecture (Figure S8).

A second limitation of the system, which can also contribute to the relatively low silicification efficiency, is that the phase separating polymers do not possess the optimal properties for silicification. Our choice of polymers rises primarily from the experimental need for macroscopic phase separation, but it is very different from biogenic silica-associated polymers.^35^ For example, the type of amine functionality, the length of the polymers, or the charge density, are all important chemical factors that can be varied. It is plausible that the use of bioinspired polymers that resemble long-chain polyamines (LCPAs) and negatively charged proteins will improve the efficiency of silica polymerization. However, this work might also highlight the limitation of a bioinspired approach, as many of the important chemical factors are unknown. We do not know if dense phases exist within silicifying cells, and if they do, we do not know their composition, salt content, or pH; all of these factors appear to play pivotal roles in the silicification process.

In conclusion, the conceptual framework of phase separation can be used as a guide to the study of bioinspired silica formation, and possibly other multistep mineralization processes. The phase boundary facilitates an interplay between two different chemical environments that give rise to distinct, but interconnected, chemical reactions. This can give rise to a concentration of the mineral building blocks within a specific phase, a situation that maintains constant supersaturation that is needed for the formation of metastable phases. The dynamic equilibrium between the dilute and dense phases allows for regulation of the mineralization reactions as changes to one phase are passively propagated to the other phase and affect the formation of the mineral.

## Supporting Information Available

Materials and methods section and Figures S1–S10 (PDF)
