# Supplementary material for: Polymer-Rich Dense Phase Can Concentrate Metastable Silica Precursors and Regulate Their Mineralization
Source: ACS Biomater Sci Eng. 2023 Feb 1;9(2):601–7. doi: 10.1021/acsbiomaterials.2c01249 (PMC9930081; doi:10.1021/acsbiomaterials.2c01249)
Supplement: Supplementary file 1 — ab2c01249_si_001.pdf [file ab2c01249_si_001.pdf]

Supplementary Information

A polymer-rich dense phase can concentrate metastable silica precursors and regulate their mineralization

Hang Zhai[a], Yuke Fan[b], Wenjun Zhang[b], Neta Varsano[c], and Assaf Gal[a]*

Pages S2-S3 – Materials and methods

Pages S4-S10 – Figure S1-S10

Materials and methods

Reagents

Poly(acrylamide-co-acrylic acid) (Mw ~520,000) and polyethylenimine (Mw ~750,000) were purchased from Sigma-Aldrich and used without any pretreatments. All reported polymer concentrations were used as the concentration of functional groups, which were calculated using the reported polymer purities and average sizes. Sodium silicate solution ($(NaOH)_x(Na2SiO3)_y{\cdot}_zH2O$, 27% SiO_2) from Sigma-Aldrich was used as the silicon source. Ultra-pure water (Milli-Q IQ 7003 Ultrapure Lab Water System, Merck) was used for solution preparation.

Polymer phase separation and silicification

0.25 mL PEI stock solution (200 mM, pH 5.0) was mixed with 0.25 mL PAMcoAA stock solution (200 mM, pH 5.0) and 0.5 mL milli-Q water to reach 1 mL. After 30 min the mixed solution was centrifuged at 10000 g for 3 min. Dilute phases were removed by pipette and the polymer dense phases were moved into plastic dishes for further silicification. To silicify the coacervates, 5 mL $Si(OH)_4$ and 10 mM PEI solution was added into the dish as a new dilute phase. The dilute phase were refreshed every 12 h.

Dynamic light scattering

Zetasizer Nano ZSP (Malvern Instruments, United Kingdom) equipped with a 633 nm laser was used for dynamic light scattering (DLS) experiments to measure particle sizes in real-time. The particle sizes were determined by intensity distribution and presented as the average values of three replicate measurements.

Light microscope imaging

Condensates were observed before and after silicification by a light microscope (Nikon Eclipse Ni-U). To track the silica within coacervates, PDMPO [2-(4-pyridyl)-5-((4-(2-dimethylaminoethyl-amino carbamoyl)methoxy)-phenyl)oxazole] (ThermoFisher Scientific, USA) was added to a final concentration of 330 µM and its fluorescence was monitored by an epifluorescence microscope.

Silica quantification

Condensates were dissolved in 5 mL 1 M NaOH. After 24 h incubation, 100 µL were further diluted to 5 mL using milli-Q water and the silica concentration was subsequently determined using the silicate test kit (Merck Millipore, USA). The amount of silica was calculated by the measured Si-concentration × 5 mL and presented as the average values of three replicate measurements.

Thermogravimetric Analysis

Lyophilized samples were analyzed by thermal gravimetric analysis (SDT Q600, TA Instruments, USA). Analyses were performed under air atmosphere (injection rate of 100 mL/min) with a heating rate of 10 K/min. When temperature approached 800 ℃, all organic parts were combusted and dry Si-contents were calculated. The dry Si-contents present in this work were present as the average values of three replicate measurements.

SEM observations

Lyophilized samples were mounted onto SEM holders. The samples were coated with 5 nm iridium (Compact Coating Unit, CCU-010, Safematic), and imaged by SEM (Sigma, Zeiss) under 5 kV. Elemental analyzes were performed by Energy-dispersive x-ray spectroscopy (EDS) using an acceleration voltage of 5 kV and the signal from the sample was recorded using a Bruker Quantax microanalysis system equipped with an XFlash6 60-mm detector.

Real-time in situ Raman monitoring

Confocal Raman Microscope (LabRam HR Evolution, Horiba) was performed to monitor Si-species in solutions and coacervates. To observe the samples, we used the ×50 objective (Olympus, LMPlanTL N) and 100 µm confocal hole. The spectra were obtained by using a laser at 532 nm as the excitation source with calibration by the characteristic band of silicon at 520.7 cm^{-1} and 600 lines/mm were set up to simultaneously scan a range of frequencies. The collected Raman data were analyzed by LabSpec 6 software.

Statistical analysis

The values of direct measurements are presented as average $\pm$ standard deviation collected from three independent repeats.

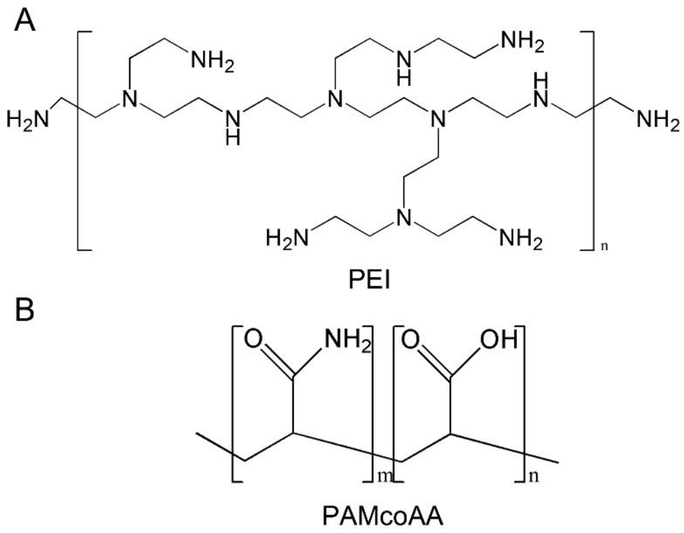

Figure S1. Structures of polyelectrolytes used in the present research. (A) Polyethylenimine (PEI), (B) poly(acrylamide-co-acrylic acid) (PAMcoAA).

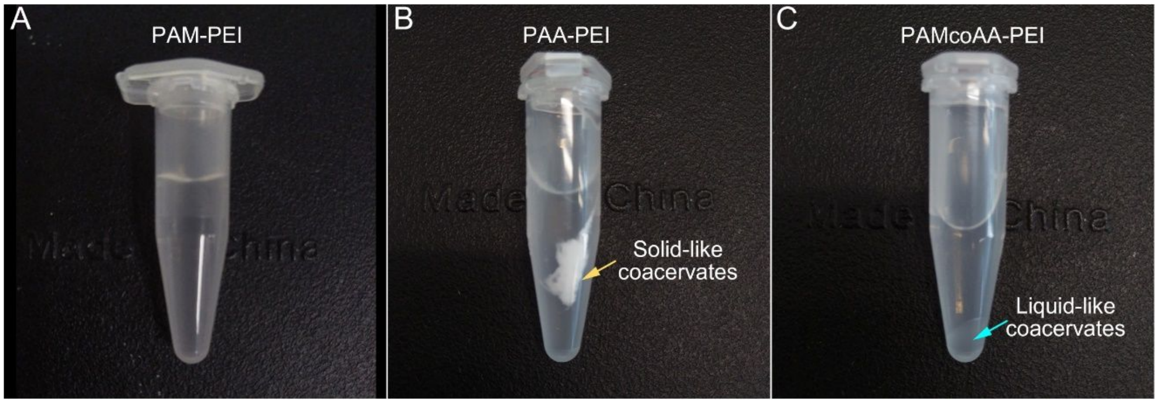

Figure S2. Photographs of products after reactions of different polymer pairs. Yellow and blue arrows in B and C indicate solid-like and liquid-like condensates, respectively.

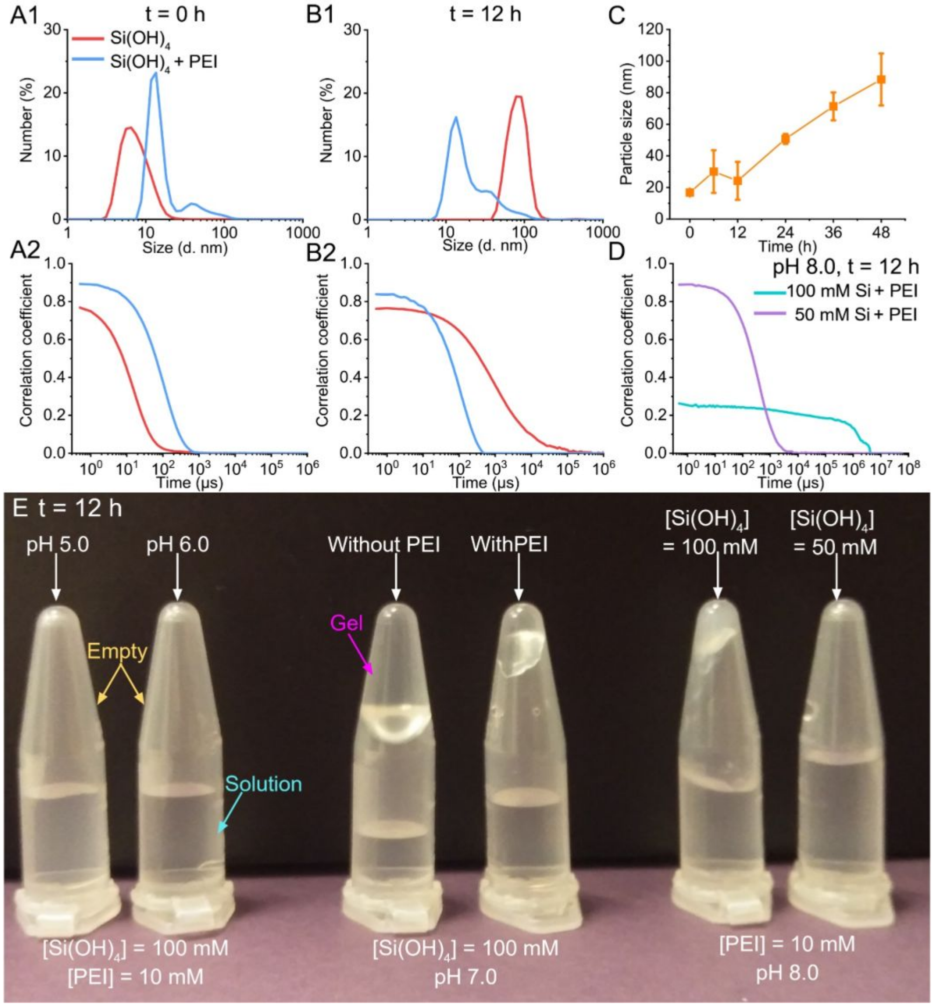

Figure S3. Stabilization of Si from gelation by PEI. Distributions of particle sizes detected by DLS measurements in 100 mM Na_2SiO_3 solutions (red) without and (blue) with 10 mM PEI at pH 5.0 (A) before and (B) after 12 h reaction. Corresponding correlation coefficients are shown below. (C) Particle sizes as a function of time showing the presence of PEI could stabilize Si for at least 12 h at pH 5.0. (D) At pH 8.0, gelation occurred causing the correlation coefficient below 0.3 but lowering Si concentration to 50 mM stabilize the solution from gelation. (E) Photographs of Si-containing solutions after 12 h reactions under different conditions show PEI can inhibit gel formation at pH 5 and 6. To achieve a gel-inhibitory effect at pH 8.0, we lowered Si-concentration to 50 mM.

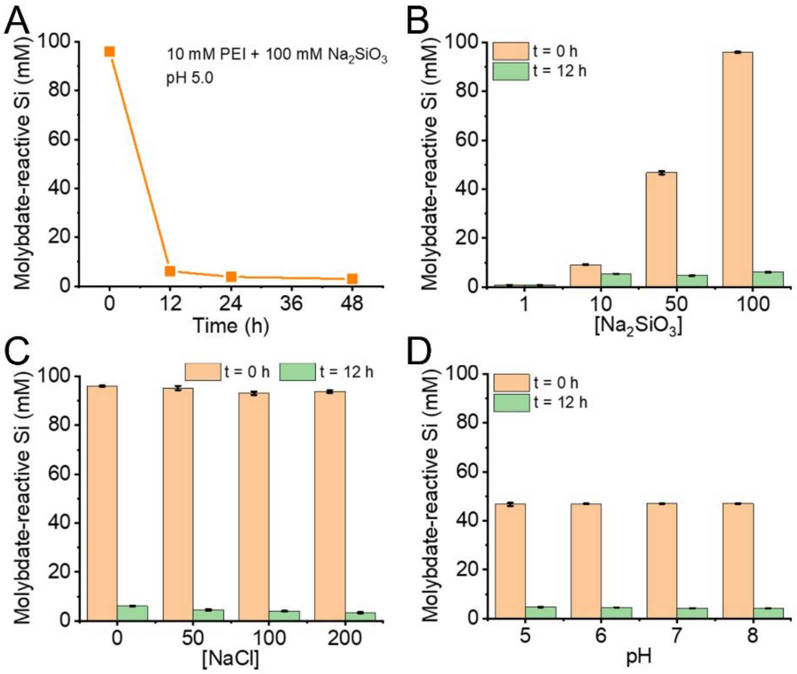

Figure S4. The concentration of mono- and di-silicic acid (the molybdate reactive silica species in solution) in the PEI-stabilized dilute phases. A) following the silicic acid concentration with time shows that even though the initial concetration is similar to the nominal concentrauon of the sodium silicate, very fast oligomers form and the silicic acid concnetration drops to few mM. B-D) the silicic acid concnetrations in the various dilute phases used in the experiments, measured both as prepared and after 12 hours. Also here the concentration decreases due to the formation of oligomers.

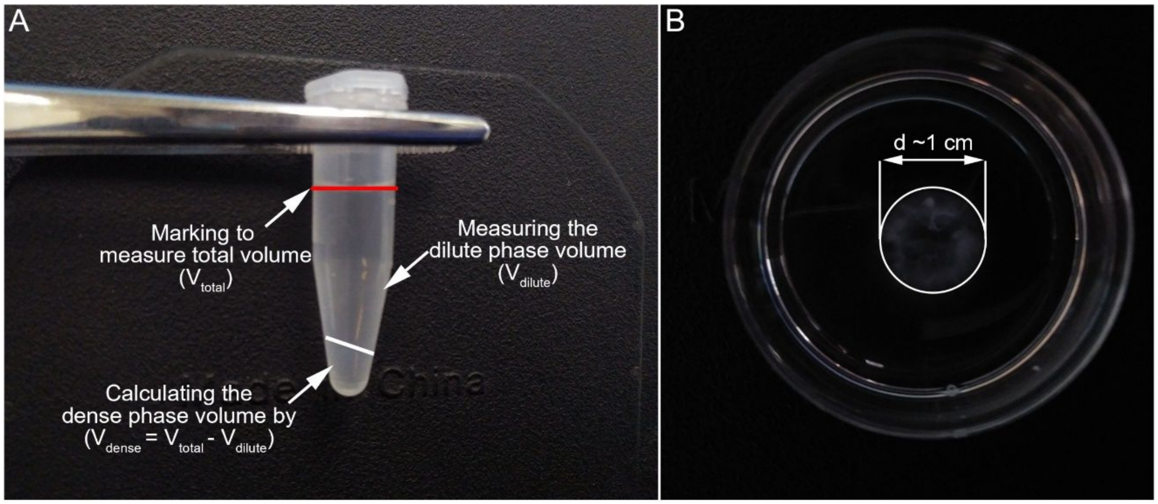

Figure S5. Steps to measure the (A) volume and (B) thickness of PEI-PAMcoAA coacervates. (A) The liquid-air interface was marked to measure the total volume (V_{total}) of the dilute phase and dense phase. The dilute phase was taken out by pipettes and got its volume (V_{dilute}) measured. The volume (V_{dense}) of the dense phase was calculated by $V_{dense} = V_{total} - V_{dilute}$. (B) The diameter, as well as the surface area, of coacervates were measured and the thickness can be obtained by V_{dense}/Surface area.

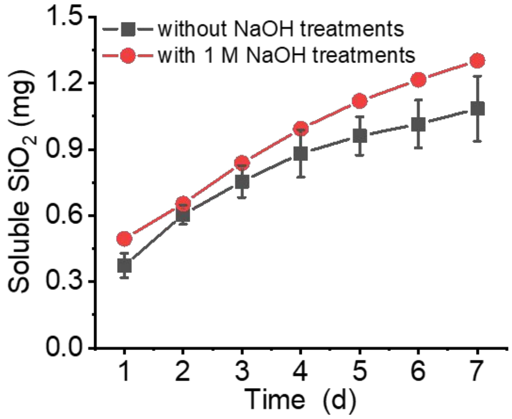

Figure S6. The amount of soluble silicic acid in the eluted dilute phase (as reported in Figure 3 A,B) was detected with the molybdate colorimetric test with and without addition of a strong base. The addition of the base, which dissolve any silica oligomers, only resulted in minor increase of detected silicic acid, demonstrating that most of the eluted 'Si' was in the form of silicic acid.

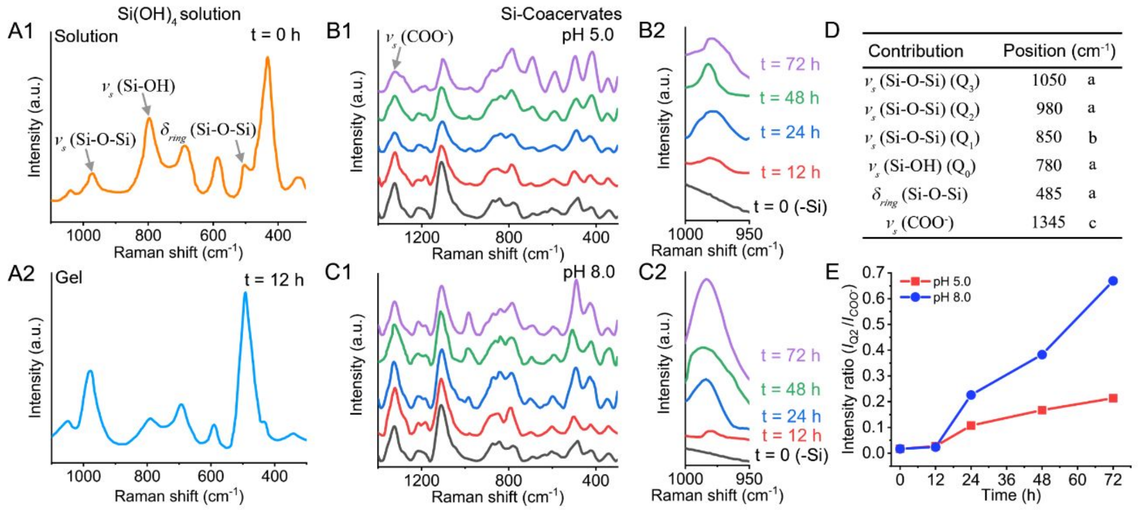

Figure S7. *In situ* real-time monitoring Si-species. Pure $Si(OH)_4$ Raman spectra collected from (A1) freshly prepared 100 mM Na_2SiO_3 (pH 5.0) solution and (A2) Si-gel after 12 h reaction. The typical peaks we focused on in the present work are marked by arrows. The time sequences of $Si(OH)_4$ Raman spectra show the intensity of v_s(Si-O-Si) got increased after gelation. Raman spectra collected from PAMcoAA-PEI-Si coacervates at pH (B1) 5.0 and (C1) 8.0. B2 and C2 are magnified regions of 950 to 1000 cm^{-1} corresponding to v_s(Si-O-Si) at 980 cm^{-1}. (D) Summarized Si-O-related Raman data from the published paper. Lowercase characters of a, b, and c in D indicate the Ref; respectively. (E) Intensity ratios of $I_{vs(Si\text{-}O\text{-}Si)\ (Q2)}$ to $I_{vs(coo\text{-})}$ suggest that increasing pH from 5.0 to 8.0 can promote the polymerization of $Si(OH)_4$.

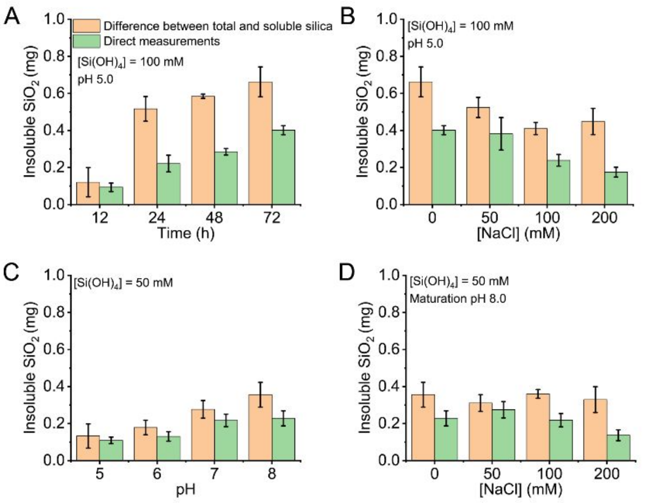

Figure S8. A) The amount of insoluble SiO_2 calculated as the difference between the total and soluble silica compared to direct measurements after various silicification times. **B**) The amount of insoluble silica as a function of ionic strength in the dilute phase at pH 5 after 72 h-silicification. **C**) The amount of insoluble silica after silicification with various pH changes. **D**) The amount of insoluble silica at pH 8 as a function of ionic strength in the dilute phase.

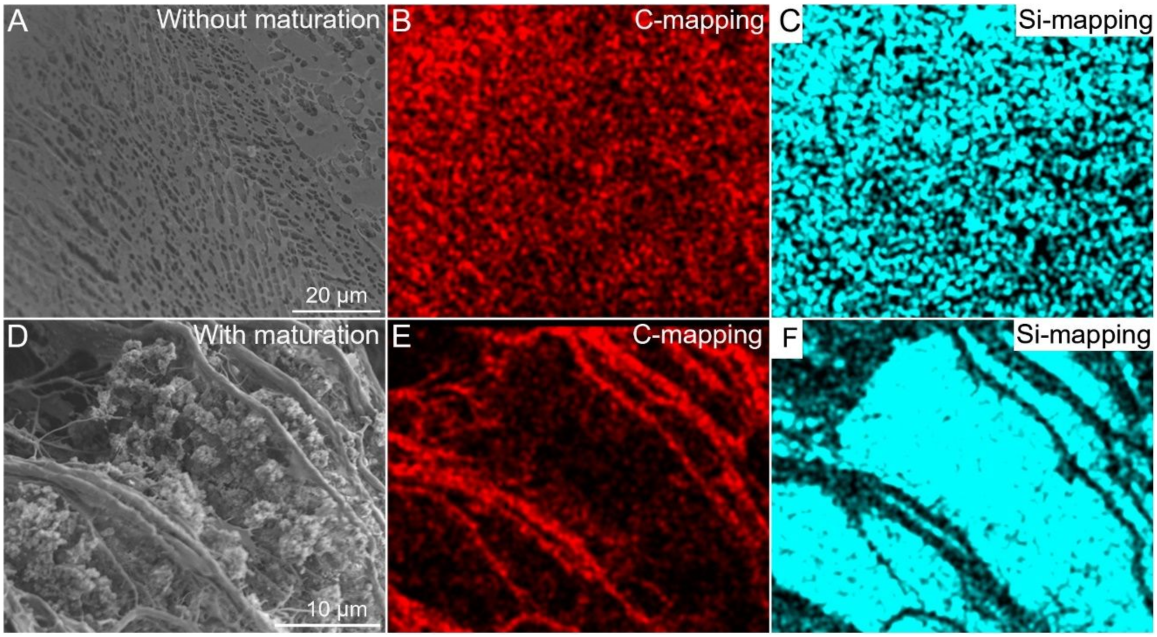

Figure S9. SEM images and corresponding C/Si-elements distribution. (A-C) Without maturation, PAMcoAA-PEI coacervates gave Si-networks with Si uniform distribution. (D-F) After maturation, silica granules formed within the coacervates.

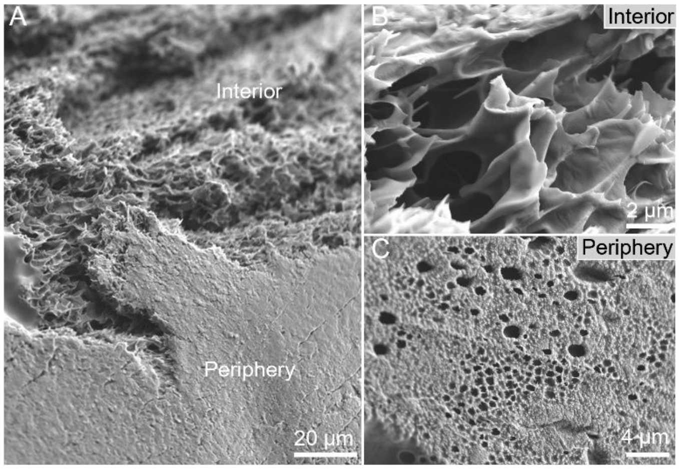

Figure S10. SEM images of lyophilized PEI-PAMcoAA coacervates showing that the thickness was large enough to expose morphological differences between the dense phase periphery and interior.
